# Polymeric nanocomposites loaded with fluoridated hydroxyapatite Ln^3+^ (Ln = Eu or Tb)/iron oxide for magnetic targeted cellular imaging

**DOI:** 10.7497/j.issn.2095-3941.2015.0014

**Published:** 2015-09

**Authors:** Jie Pan, Wei-Jiao Liu, Chao Hua, Li-Li Wang, Dong Wan, Jun-Bo Gong

**Affiliations:** ^1^State Key Laboratory of Hollow Fiber Membrane Materials and Processes, School of Environmental and Chemical Engineering, Tianjin Polytechnic University, Tianjin 300387, China; ^2^Key Laboratory of Green Process and Engineering, Institute of Process Engineering, Chinese Academy of Science, Beijing 100190, China; ^3^Department of Chemical Engineering, School of Chemical Engineering and Technology, Tianjin University, Tianjin 300072, China

**Keywords:** Cancer, cellular imaging, nanocomposites, magnetic targeted, hydroxyapatite (HAP) doped with rare earth

## Abstract

**Objective:**

To fabricate polymeric nanocomposites with excellent photoluminescence, magnetic properties, and stability in aqueous solutions, in order to improve specificity and sensitivity of cellular imaging under a magnetic field.

**Methods:**

Fluoridated Ln^3+^-doped HAP (Ln^3+^-HAP) NPs and iron oxides (IOs) can be encapsulated with biocompatible polymers via a modified solvent exaction/evaporation technique to prepare polymeric nanocomposites with fluoridated Ln^3+^-HAP/iron oxide. The nanocomposites were characterized for surface morphology, fluorescence spectra, magnetic properties and *in vitro* cytotoxicity. Magnetic targeted cellular imaging of such nanocomposites was also evaluated with confocal laser scanning microscope using A549 cells with or without magnetic field.

**Results:**

The fabricated nanocomposites showed good stability and excellent luminescent properties, as well as low *in vitro* cytotoxicity, indicating that the nanocomposites are suitable for biological applications. Nanocomposites under magnetic field achieved much higher cellular uptake via an energy-dependent pathway than those without magnetic field.

**Conclusion:**

The nanocomposites fabricated in this study will be a promising tool for magnetic targeted cellular imaging with improved specificity and enhanced selection.

## Introduction

With the advances in molecular and cellular techniques, research about the diagnosis of disease has been directed toward the underlying molecular and genomic aberrations, rather than toward clinical signs and symptoms alone. Molecular imaging is a developing research discipline aimed at visually characterizing normal and pathologic processes in living organisms at the cellular and molecular levels^1^^-^^4^. Modalities used in molecular imaging includes positron emission tomography, magnetic resonance imaging, single-photon emission computed tomography, optical imaging, and ultrasound^5^. These modalities differ in terms of spatial resolution, temporal resolution, sensitivity in probe detection, depth of signal penetration, availability of biocompatible molecular imaging agents, and cost. Optical imaging, an emerging molecular imaging mode, is based on the detection of photons after their interaction with the tissue. This technique has high sensitivity and spatial resolution, features fast and easy to perform procedures, and requires inexpensive and available optical reporters and dyes for simultaneous imaging of multiple processes^3^. As an optical approach, fluorescence imaging is used for molecular imaging.

As hydroxyapatite (HAP) is the main inorganic component of bones and teeth of humans and animals, it exhibits good biocompatibility in biological applications^6^^-^^8^. HAP doped with rare earth elements has been used as luminescent materials for biological imaging^8^^-^^17^. Hui *et al*.^16^ used a hydrothermal approach to dope rare earth elements with fluorine ions to fabricate fluoridated HAP nanocrystals, which demonstrated excellent luminescent properties for biological imaging. The quenching of the excited state of rare earth elements is caused by −OH ions in the lattice, which were replaced with F^−^ ions to prepare materials with low vibrational energies for rare earth fluorescent transition^8^. Hui *et al*.^16^ described doped HAP nanoparticles (NPs) as fluorescing probes with excellent biocompatibility, good biodegradability, and prominent luminescent features. Compared to traditional fluorescent dyes, doped HAP NPs exhibit high fluorescence intensity, enhanced photostability, improved stability, and high resistance to photobleaching. Quantum dots (QDs) have been widely used as luminescence probes. QDs have various advantages, including tunable emission from visible to infrared wavelengths by changing their size and composition, broad excitation spectra because of high absorption coefficients, high quantum yield of fluorescence, strong brightness, photostability, and high resistance to photobleaching^18^^-^^20^. Although QDs are suitable for biological imaging, they exhibit toxicity caused by the oxidative degradation of their heavy metal contents, thereby limiting their applications in biological fields^21^^-^^23^. The toxicity of QDs can only be partly resolved through surface modification or encapsulation in biodegradable NPs^24^^-^^29^. As an alternative to QDs, fluoridated HAP:Ln^3+^ NPs present lower toxicity; these NPs are also inexpensive and feature anti-corrosion properties. Therefore, fluoridated HAP:Ln^3+^ NPs can be potentially used for molecular imaging.

Targeted molecular imaging of cancer is generally achieved through passive and active targeting^30^^-^^33^. In passive targeting, probes accumulate at tumor sites through an enhanced permeability and retention effect, which can be attributed to two factors^34^^-^^36^. First, angiogenic tumors produce vascular endothelial growth factor, which hyperpermeabilizes tumor-associated neovasculature and results in the leakage of circulating probes. Second, tumors lack an effective lymphatic drainage system, which causes subsequent probe accumulation^33^. In active targeting, targeting agents, such as ligands, attach on the surface of small particles through various conjugation mechanisms^2^^,^^33^^,^^37^^,^^38^. The targeted probes can recognize and bind to specific ligands unique to cancer cells; subsequently, the probes largely accumulate in cancer cells while minimizing distribution in non-cancerous cells adjacent to the targeted tissue. Although active targeting is superior in molecular imaging, it is limited for clinical applications because of patient-to-patient variation in receptor expression levels and non-specific expression of receptors in normal tissues. Unlike active targeting, magnetic targeting utilizes magnetic field to accumulate magnetic probes in the cellular area where the magnet is placed^39^^-^^47^. Magnetic targeting is a general targeting approach because it is based on physical interactions and not limited by specific receptor expression. Gu *et al*.^46^ fabricated magnetic mesoporous silica nanoparticles (M-MSNs) and found that the internalization of M-MSNs by A549 cancer cells could be accelerated and enhanced under magnetic field; the internalization is mainly through an energy-dependent pathway, namely, clathrin-induced endocytosis, rather than passive diffusion or magnetic pull-down process.

**Figure 1:**
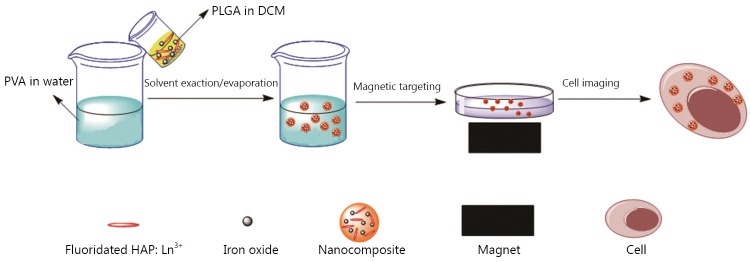
Scheme of the synthesis and applications of the fluoridated Ln^3+^-HAP/IOs PLGA nanocomposites.

In this study, a facile method was used to fabricate novel nanocomposites for magnetic targeted cellular imaging of cancer. Fluoridated Ln^3+^-doped HAP (Ln^3+^-HAP) NPs and iron oxides (IOs) can be encapsulated with biocompatible polymers [such as poly(D,L-lactide-co-glycolide), PLGA] via a modified solvent exaction/evaporation technique to prepare polymeric nanocomposites with fluoridated Ln^3+^-HAP/iron oxide. The fabricated composite can be used to improve specificity and sensitivity of cellular imaging through magnetic targeting. In this study, an external magnetic field was used for targeting to allow the nanocomposites to be as close as possible to A549 cancer cells. Most nanocomposites around A549 cells can be internalized by the cells via an energy-dependent pathway. This study utilizes magnetic field to achieve targeted cellular imaging of cancer with fluoridated Ln^3+^-HAP NPs for cancer diagnosis. The scheme of synthesis and applications of fluoridated Ln^3+^-HAP/IOs PLGA nanocomposites is presented in **Figure 1**.

## Materials and methods

### Materials

Ca(NO_3_)_2_·4H_2_O, Na_3_PO_4_·12H_2_O, NaF, NaOH, octadecylamine, oleic acid, ethanol, cyclohexane, FeSO_4_·(NH_4_)_2_SO_4_·6H_2_O, dichloromethane (DCM), and PVA (MW: 30,000-70,000) were obtained from Beijing Chemical Reagents Company (China). Eu(NO_3_)_3_·6H_2_O, Tb(NO_3_)_3_·6H_2_O, and PLGA (L:G molar ratio: 75:25, MW: 100,000-130,000) were purchased from Sigma (St. Louis, MO, USA). Dulbecco’s modified Eagle’s medium (DMEM), antibiotics (penicillin-streptomycin solution), Triton X-100, 3-(4,5-dimethylthiazol-2-yl)-2,5-diphenyl-tetrazolium bromide (MTT), diethyl ether, chloroform, and cholesterol were purchased from Sigma-Aldrich (St. Louis, MO, USA). Fetal bovine serum (FBS) was purchased from Gibco (Life Technologies, AG, Switzerland). Millipore water was produced using the Milli-Q Plus System (Millipore Corporation, Bedford, USA).

### Preparation of fluoridated Ln^3+^-HAP (Ln = Eu or Tb) NPs

Fluoridated Ln^3+^-HAP NPs were prepared using the protocol described in the literature^8^. Briefly, octadecylamine (0.5 g) were dissolved in oleic acid, (4 mL) in a Teflon-lined autoclave (50 mL). The solution was mixed with ethanol (16 mL) and an aqueous solution of Ca(NO_3_)_2_ (0.28 M, 7 mL) under agitation. The solution was then added with aqueous solutions of Eu(NO_3_)_3_ or Tb(NO_3_)_3_ (0.28 M, 0.7 mL), NaF (0.28 M, 1.4 mL), and Na_3_PO_4_ (0.16 M, 7 mL). The mixture was agitated for 5 min, sealed, and hydrothermally treated at 180 °C for 12 h. The obtained fluoridated Ln^3+^-HAP NPs were collected through centrifugation and washed several times with cyclohexane and ethanol.

### Preparation of IOs

IOs were fabricated according to the method reported in the literature^48^. Briefly, 0.6275 g of FeSO_4_·(NH_4_)_2_SO_4_·6H_2_O was dissolved in 16 mL of water. The solution was mixed with 0.8 g of NaOH, 8 mL of oleic acid and 8 mL of ethanol under stirring at room temperature. The solution was then added with an aqueous solution of FeSO_4_·(NH_4_)_2_SO_4_·6H_2_O. The mixed reactants were then transferred to a 50 mL sealed autoclave and heated at 180 °C for 10 h. The products were collected through centrifugation and washed at least 3 times with cyclohexane and ethanol. Finally, IOs were obtained through drying and stored for further use.

### Preparation of fluoridated Ln^3+^-HAP/IOs PLGA nanocomposites

Fluoridated Ln^3+^-HAP/IOs PLGA nanocomposites were prepared through a modified solvent exaction/evaporation technique. Briefly, 30 mg of the mixture of fluoridated Ln^3+^-HAP NPs and IOs (2:1, 1:1, and 1:2; mass ratio) were dissolved in 4 mL of DCM containing 50 mg of PLGA. The solution was added to 60 mL of aqueous solution with 300 mg of PVA. The mixture was then sonicated for 100 s at 75 W. The emulsion was agitated overnight to evaporate DCM. The formed suspension was centrifuged with deionized water. Finally, fluoridated Ln^3+^-HAP/IOs PLGA nanocomposites were obtained through centrifugation.

### Characterization

The morphologies of the nanocomposites were observed with a HITACHI H-7650B transmission electron microscope (TEM) at 100 kV and a FEI Tecnai G2 F20 S-Twin high-resolution TEM at 200 kV. The nanocomposite suspension was dropped on the surface of copper grid with carbon film and dried at room temperature. The fluorescence spectra of the nanocomposites were recorded using a HITACHI F-4500 fluorescence spectrophotometer with an excitation wavelength of 405 and 488 nm for the fluoridated Eu^3+^-HAP/IOs PLGA nanocomposites and fluoridated Tb^3+^-HAP/IOs PLGA nanocomposites, respectively. The magnetic properties of the fluoridated Ln^3+^-HAP/IOs PLGA nanocomposites were determined with a vibrating sample magnetometer (VSM) at room temperature. Dried nanocomposites of known mass were placed in non-magnetic aluminum sheet and then subjected to varied magnetic fields that ranged from −2×10^4^ to 2×10^4^ Oe.

### Cell experiments

#### Cell culture

Human cervical HeLa cell line A549 were obtained from the American Type Culture Collection. All cell culture related reagents were purchased from Invitrogen. The cells were grown in DMEM with 10% FBS and 1% penicillin/streptomycin. The fluoridated Ln^3+^-HAP/IOs PLGA nanocomposites containing a 1:1 mass ratio of the fluoridated Eu^3+^-HAP and IOs were used for cell experiments.

#### Cytotoxicity of nanocomposites

The viability of A549 cells incubated with the nanocomposites was evaluated with MTT assay. Briefly, the cells were seeded in 96-well microplates at a density of 5×10^4^ cells/mL. After 24 h of cell attachment, the cells were incubated with 10, 20, 40, 80, 150, and 300 µg/mL nanocomposites for 8 and 24 h. The nanocomposites were then removed, and the cells were washed with PBS three times. The wells were washed twice with PBS and added with 10 μL of MTT supplemented with a culture medium. After 4 h of incubation, the culture medium was removed and the precipitate was dissolved in isopropanol. The absorbance of the wells was determined with a microplate reader (VictorШ, Perkin-Elmer) with a wavelength of 570 nm and reference wavelength of 620 nm. Cell viability was calculated with the following equation:

$$Cell\;viability\left(\%\right)=\frac{Int_{s}}{Int_{control}}\times 100\%$$

where Int_s_ is the absorbance intensity of the cells incubated with the nanocomposites suspension and Int_control_ is the absorbance intensity of the cells incubated with the incubation medium only (positive control).

#### Magnetic targeted cellular imaging

A549 cells were maintained at 37 °C in a culture medium under a humidified condition of 5% CO_2_. On the day prior to treatment, the cells were seeded in a glass bottom dish with a density of 50,000 cells/mL. For magnetic targeted cellular imaging, a cubic permanent magnet (1.3T) was placed under the edge of the dish and the cells were incubated with 250 µg/mL nanocomposites for 2 h at 37 °C. Control groups were incubated with the nanocomposites at the same concentration for 2 h without the magnetic field. The cells were then washed with PBS, and the nuclei were stained with 4',6-diamidino-2-phenylindole dihydrochloride for 30 min. The stained cells were washed twice with PBS, and cell images were obtained using a confocal laser scanning microscope (CLSM, Zeiss 710 3-channel; Zeiss, Germany) with an excitation wavelength of 405 nm.

## Results and discussion

### Charactarization of fluoridated Ln^3+^-HAP/IOs PLGA nanocomposites

#### Surface morphology

**Figure 2:**
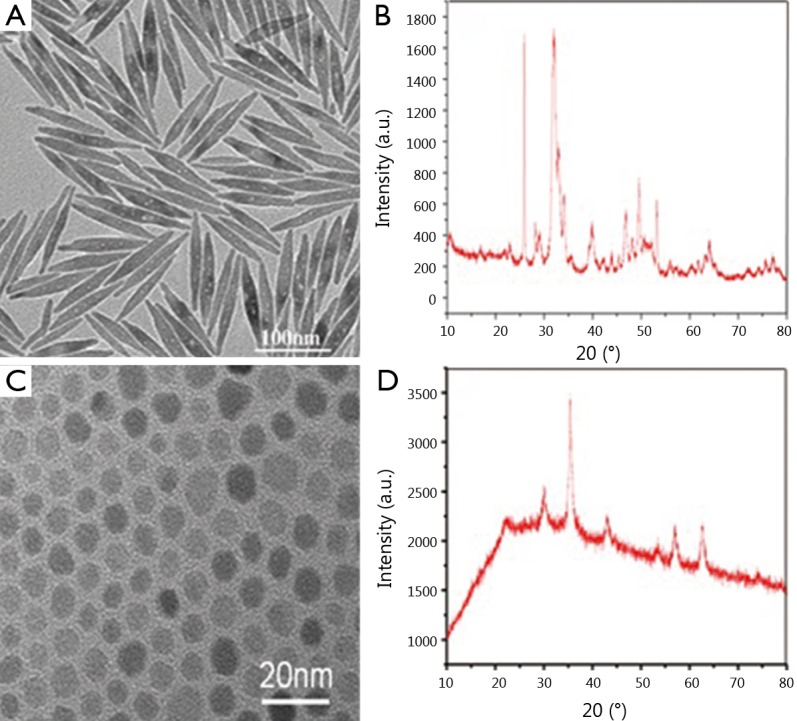
(A) TEM image of hydrophobic fluoridated Eu^3+^-HAP NPs. (B) TEM image of hydrophobic IOs. (C) XRD patterns of fluoridated Eu^3+^-HAP NPs. (D) XRD patterns of IOs. TEM, transmission electron microscope; HAP, hydroxyapatite; NP, nanoparticle; IO, iron oxide.

**Figure 3:**
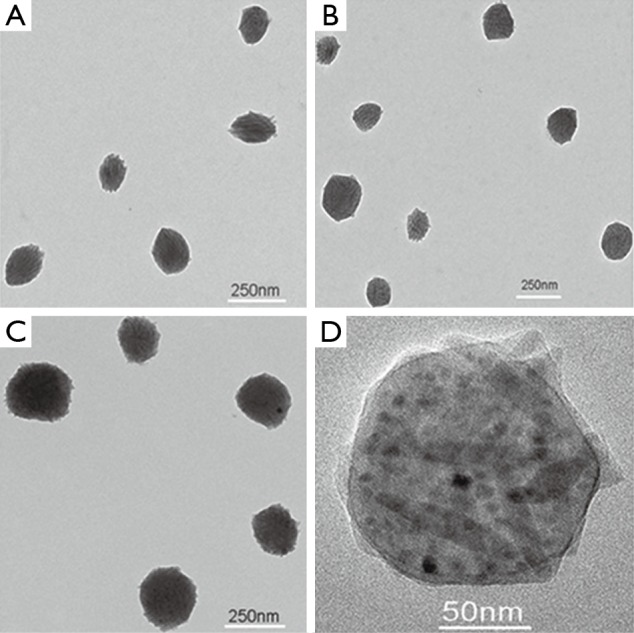
TEM images of the fluoridated Eu^3+^-HAP/IOs PLGA nanocomposites under different mass ratios of fluoridated Eu^3+^-HAP and IOs. (A) 2:1. (B) 1:1. (C) 1:2. (D) HTEM image of the fluoridated Ln^3+^-HAP/IOs PLGA nanocomposites at 1:1 mass ratio of fluoridated Eu^3+^-HAP and IOs. TEM, transmission electron microscope; HAP, hydroxyapatite; IO, iron oxide.

**Figure 4:**
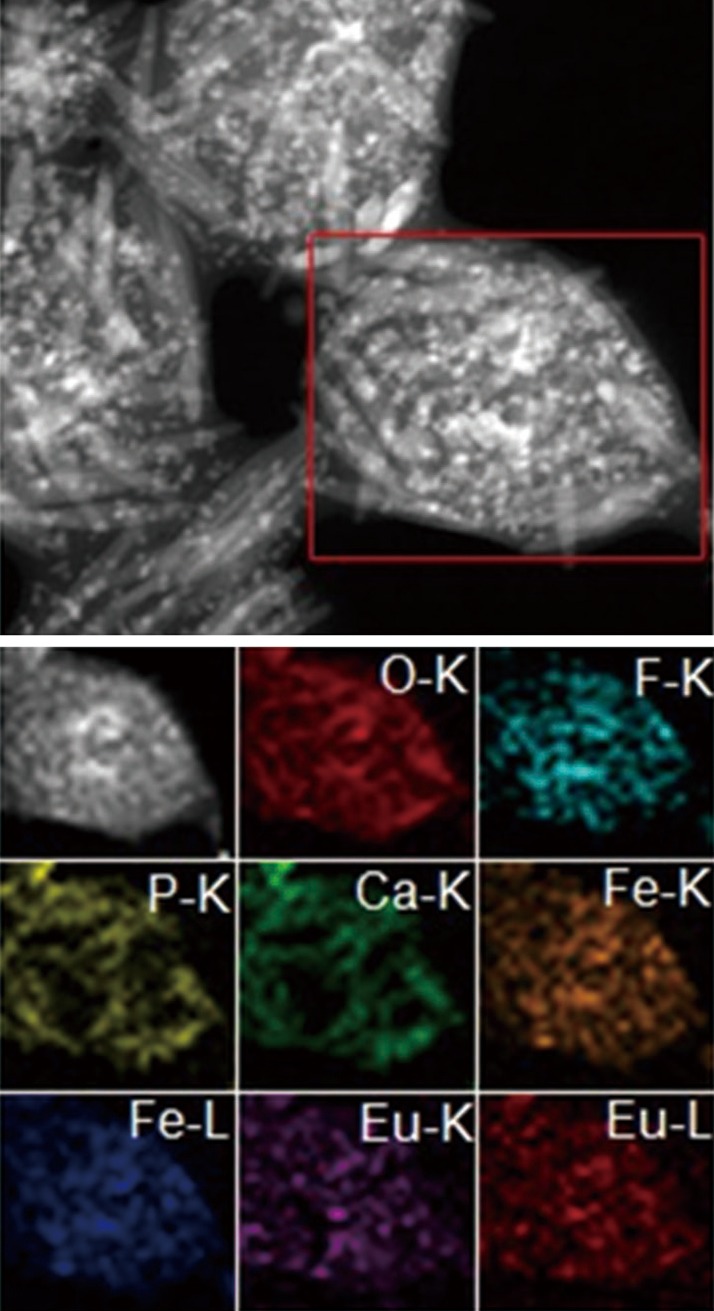
EDS map of the fluoridated Eu^3+^-HAP/IOs PLGA nanocomposites. HAP, hydroxyapatite; IO, iron oxide.

The TEM images of fluoridated Eu^3+^-HAP NPs and IOs are shown in **Figure 2A,B**. The length of the fluoridated Eu^3+^-HAP NPs is about 100 nm, the size of IOs is around 8 nm, and both are well monodispersed. **Figure 2C,D** represent the XRD patterns of fluoridated Eu^3+^-HAP NPs and IOs, respectively. The XRD image of fluoridated Eu^3+^-HAP NPs shows the absence of impurities in the final products, thereby demonstrating that Eu^3+^ has been successfully doped into HAP. The XRD image of IOs displays that the final product is a pure phase of IOs. **Figure 3A-C** show the TEM images of the fluoridated Eu^3+^-HAP/IOs PLGA nanocomposites with different Eu^3+^-HAP and IOs mass ratios (2:1, 1:1, and 1:2). These nanocomposites are dispersed as individual nanocomposite with a uniform size of about 200 nm in diameter under each ratio. **Figure 3D** displays the high-resolution TEM image of the nanocomposite at 1:1 mass ratio of fluoridated Eu^3+^-HAP and IOs. Fluoridated Eu^3+^-HAP and IOs can be well encapsulated into PLGA NPs, and the encapsulation does not result in morphological changes. The EDS map in **Figure 4** presents the element distribution of the fluoridated Eu^3+^-HAP/IOs PLGA nanocomposites, thereby demonstrating that fluoridated Eu^3+^-HAP and IOs can be well encapsulated into PLGA NPs. The nanocomposites with fluoridated Eu^3+^-HAP and IOs at a mass ratio of 1:1 were used for the following study because of their fluorescence and magnetic property.

#### Emission spectrum

**Figure 5:**
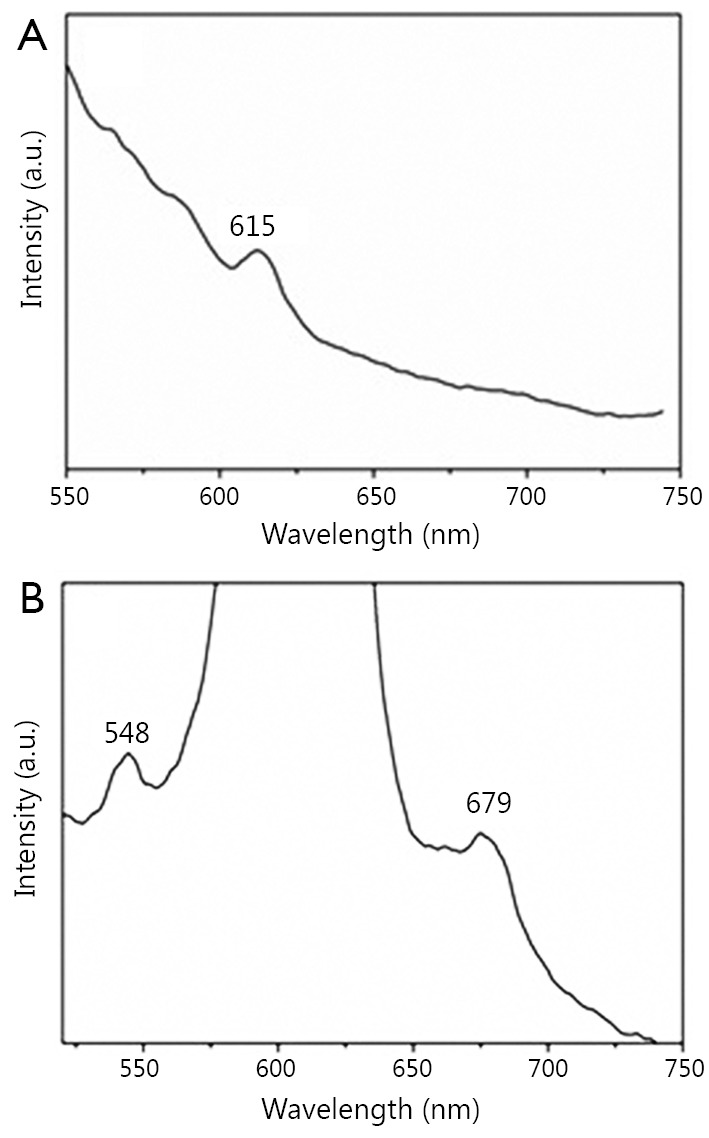
(A) Luminescent spectrum of the fluoridated Eu^3+^-HAP/IOs PLGA nanocomposites under excitation at 405 nm. (B) Luminescent spectrum of the fluoridated Tb^3+^-HAP/IOs PLGA nanocomposites under excitation at 488 nm. HAP, hydroxyapatite; IO, iron oxide.

The emission spectra of the fluoridated Eu^3+^-HAP/IOs PLGA nanocomposites and fluoridated Tb^3+^-HAP/IOs PLGA nanocomposites in water are demonstrated in **Figure 5**. The emission spectrum of the fluoridated Eu^3+^-HAP/IOs PLGA nanocomposites was determined from 550 to 750 nm under 405 nm excitation (**Figure 5A**), and the main emission peak of nanocomposites is located at 615 nm. **Figure 5B** shows the emission spectrum of the fluoridated Tb^3+^-HAP/IOs PLGA nanocomposites under 488 nm exitation, and the main emission peak is located at 548 nm. The spectra of the fluoridated Ln^3+^-HAP/IOs PLGA nanocomposites demonstrate that the strong fluorescence of the nanocomposites remains, despite the encapsulation of fluoridated Ln^3+^-HAP NPs into PLGA NPs. As the nanocomposites are stable with the existence of IOs, they are considered suitable for targeted cellular imaging.

#### Magnetic property

**Figure 6:**
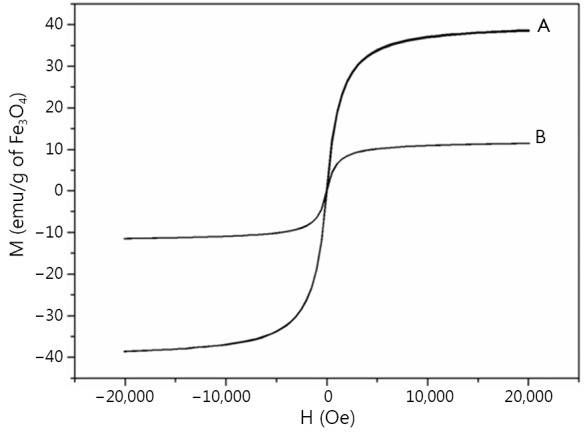
Hysteresis curve at room temperature. (A) Hydrophobic IOs NPs. (B) Fluoridated Eu^3+^-HAP/IOs PLGA nanocomposites. IO, iron oxide; NP, nanoparticle; HAP, hydroxyapatite.

The magnetic properties of IOs and the fluoridated Eu^3+^-HAP/IOs PLGA nanocomposites were obtained with a VSM. The hysteresis M–H curves are shown in **Figure 6**. Saturation magnetizations are 38.4 and 11.5 emu/g for IOs and the nanocomposites, respectively. The decrease in saturation magnetization for the nanocomposites may be due to the encapsulation of the PLGA matrix. .

### *In vitro* cytotoxicity of nanocomposites

**Figure 7:**
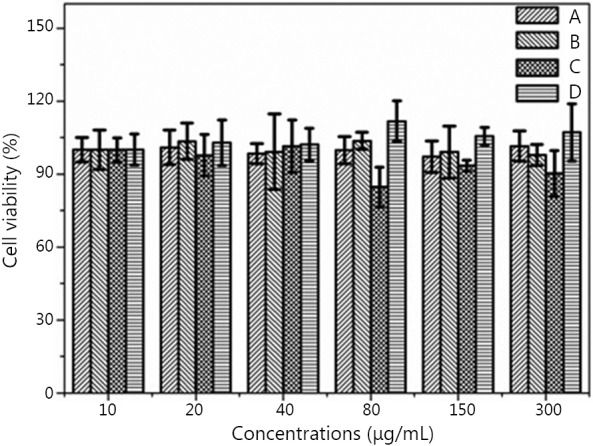
Cell viability of A549 cells at 37 °C under different nanocomposite concentrations. (A) Fluoridated Eu^3+^-HAP/IOs PLGA nanocomposites cultured for 8 h. (B) Fluoridated Tb^3+^-HAP/IOs PLGA nanocomposites cultured for 8 h. (C) Fluoridated Eu^3+^-HAP/IOs PLGA nanocomposites cultured for 24 h. (D) Fluoridated Tb^3+^-HAP/IOs PLGA nanocomposites cultured for 24 h. HAP, hydroxyapatite; IO, iron oxide.

The cytotoxity of the fluoridated Ln^3+^-HAP/IOs PLGA nanocomposites to A549 cells was evaluated using MTT assay to examine their suitability for biological applications. **Figure 7** shows that the fluoridated Ln^3+^-HAP/IOs PLGA nanocomposites display low cytotoxicity to A549 cells. The cell viability values are higher than 90% even when the concentration of the nanocomposites reached 300 μg/mL. This finding further confirms the low toxicity of the nanocomposites. Therefore, the fluoridated Ln^3+^-HAP/IOs PLGA nanocomposites could be potentially used for biological applications.

### Magnetic targeted cellular imaging

**Figure 8:**
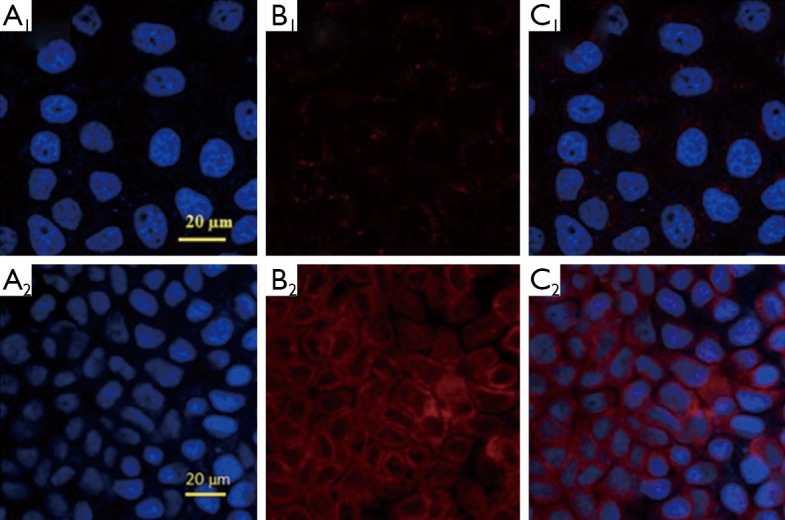
Confocal laser scanning microscopy images of A549 cells incubated with 250 µg/mL fluoridated Eu^3+^-HAP/IOs PLGA nanocomposites at 37 °C for 2 h without magnetic field (Row 1) and under a magnetic field of 1.3T (Row 2). (A) The blue channel with excitation at 340 nm. (B) The red channel with excitation at 405 nm. (C) The combined red and blue channels. HAP, hydroxyapatite; IO, iron oxide.

The application of the fluoridated Ln^3+^-HAP/IOs PLGA nanocomposites for magnetic targeted cellular imaging was investigated with CLSM with or without magnetic field. **Figure 8** demonstrates the CLSM images of A549 cells incubated with 250 µg/mL fluoridated Eu^3+^-HAP/IOs PLGA nanocomposites at 37 °C for 2 h without the magnetic field (Row 1) and under a magnetic field of 1.3T (Row 2). The CLSM images in red channel in **Figure 8** show the fluorescence images of fluoridated Eu-HAP NPs at an excitation wavelength of 405 nm. The enhanced fluoresence signals in Row 2 reveal the high cellular uptake of the fluoridated Eu^3+^-HAP/IOs PLGA nanocomposites under external magnetic field for 2 h, whereas fluoresence signals are low without external magnetic field (Row 1). The enhanced uptake of the nanocomposites by A549 cancer cells under magnetic field may result from the internalization of A549 cells via an energy-dependent pathway. These findings indicate that the fluoridated Eu^3+^-HAP/IOs PLGA nanocomposites can be efficiently used for magnetic targeted cellular imaging of cancer.

## Conclusion

Fluoridated Ln^3+^-HAP/IOs PLGA nanocomposites were prepared through a modified solvent exaction/evaporation technique. The fabricated nanocomposites show excellent photoluminescence, magnetic properties, and stability in aqueous solutions. Thus, the composites are suitable for targeted cellular imaging. The results of *in vitro* experiments further confirm that the nanocomposites exhibit low toxicity and can be successfully applied to improve the specificity and sensitivity of cellular imaging under magnetic field. The nanocomposites fabricated in this study will be a promising tool for magnetic targeted cellular imaging with improved specification and enhanced selection.
